# Dysphagia related to diffuse idiopathic skeletal hyperostosis (DISHphagia)

**DOI:** 10.1002/ccr3.2449

**Published:** 2019-09-27

**Authors:** Dhia Kaffel, Hela Kchir

**Affiliations:** ^1^ Institut National Mohammed Kassab d'Orthopedie La Mannouba Tunisia; ^2^ Hopital la Rabta Tunis Tunisia

**Keywords:** dishphagia, dysphagia, forestier's disease

## Abstract

The important clinical teaching of our case is that dysphagia most likely caused by an extradigestive pathology; hence, imaging studies of the neck is very important in the evaluation process.

## Graphical abstract

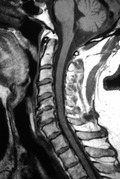

## 1 CLINICAL IMAGE

**Figure 1:**
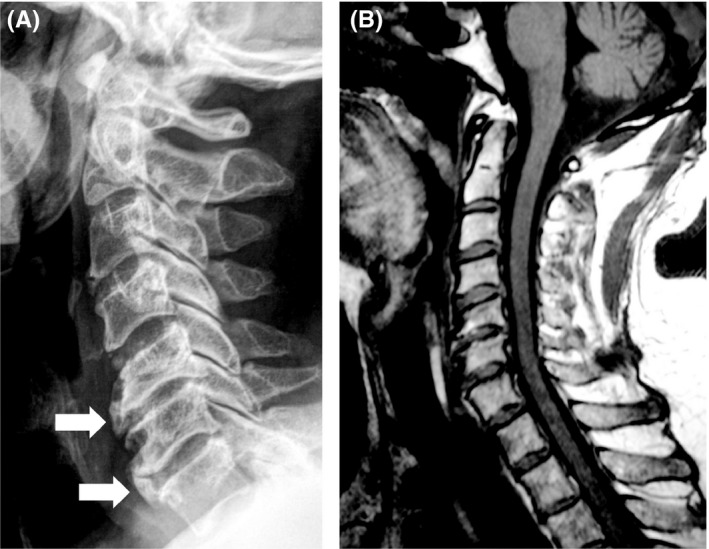
A, Plain radiograph of the cervical spine revealing a diffuse idiopathic skeletal hyperostosis with an ossification of the posterior longitudinal ligament associated to an anterior longitudinal ligament ossification (arrow). B, MRI of the cervical spine showed a compression on the adjacent wall of the esophagus. There is no spinal cord compression

A 73‐year‐old patient with Diabetes type 2, suffering from difficulty in swallowing solid food evolving since 10 months. He did not report any significant weight loss or dysphonia but since 2 years he suffered from cervical pains. The examination showed a painful and limited range of motion cervical spine. There was no neck swelling or distorted neck contour. He underwent esophagoscopy which revealed swelling of the cervical esophageal mucosa. Cervical spine’ plain radiographs (Figure 1A) and MRI (Figure 1B) showed an ossification of the posterior longitudinal ligament associated with a massive anterior longitudinal ligament ossification. MRI also revealed a compression on the adjacent wall of the esophagus. The diagnosis of a diffuse idiopathic skeletal hyperostosis (Resnick criteria) was retained.

The important clinical teaching of our case is that dysphagia is rarely isolated; it frequently reveals an underlying pathology, which can be extradigestive. MRI is very helpful tool in patients with DISHphagia.^1^

## CONFLICT OF INTEREST

None declared.

## AUTHOR CONTRIBUTIONS

DK: I submitted this manuscript. I took the pictures. I also wrote the text with the help of the co‐author. HK: The first doctor who saw the patient. She helped in making the final diagnosis. She also participated in taking pictures and writing the text.
